# 儿童急性白血病合并毛霉病6例临床分析

**DOI:** 10.3760/cma.j.issn.0253-2727.2023.07.014

**Published:** 2023-07

**Authors:** 蕾 张, 笛箫 钟, 梅 岳, 立田 玄, 朝霞 张, 娟娟 李, 君惠 李, 继珍 邹, 淯淳 闫, 嵘 刘

**Affiliations:** 1 首都儿科研究所附属儿童医院血液科，北京 100020 Department of Hematology, Capital Institute of Pediatrics, Beijing 100020, China; 2 首都儿科研究所附属儿童医院病理科，北京 100020 Department of Pathology, Capital Institute of Pediatrics, Beijing 100020, China; 3 首都儿科研究所附属儿童医院放射科，北京 100020 Department of Imaging, Capital Institute of Pediatrics, Beijing 100020, China

毛霉病是由接合菌门毛霉菌目真菌引起的感染，好发于免疫缺陷及血液肿瘤疾病患儿，死亡率高，为导致儿童白血病患者死亡的重要原因之一。据文献报道，儿童血液肿瘤性疾病患者中毛霉病的发生率为2.2％～6.9％[1]–[2]。早期识别诊断、及时启动有效抗感染治疗、手术清除感染灶为治疗成功的关键因素。本研究回顾性分析我中心确诊的6例儿童急性白血病合并毛霉病的病例资料，探讨临床特点、诊断、治疗及转归，为临床积累经验。

## 病例与方法

收集首都儿科研究所附属儿童医院血液科自2017年11月至2022年4月确诊的6例儿童急性白血病合并毛霉病患者的病例资料。其中男4例，女2例。中位年龄5.5（1～14）岁。回顾性分析临床表现、感染部位、影像学表现、治疗及转归等。诊断标准及疗效评估参考《血液病/恶性肿瘤患者侵袭性真菌病的诊断标准与治疗原则（第六次修订版）》[3]。疗效评价中有效包括完全缓解和部分缓解；无效包括稳定、疾病进展和死亡。随访截止时间为2022年8月30日。

## 结果

一、临床特征

6例患儿临床资料见表1。6例患儿均确诊为急性白血病，其中2例为急性B淋巴细胞白血病（B-ALL）,1例为急性T淋巴细胞白血病（T-ALL），1例为急性髓系白血病部分分化型（AML-M_2_），1例为急性巨核细胞白血病，1例为伯基特白血病。5例患儿均为治疗期间出现感染，2例B-ALL及1例AML-M_2_患儿均于首次诱导治疗期间发生感染，1例T-ALL及1例伯基特白血病于原发病已缓解、进行强化治疗期间发生感染，感染发生时间为化疗后1～5个月。1例急性巨核细胞白血病患儿曾接受亲缘半相合外周血造血干细胞移植，口服环孢素、激素预防移植物抗宿主病，于移植后5个月原发病复发再次接受诱导化疗时出现感染。6例患儿从出现症状至确诊时间为10～25 d，平均17 d。4例患儿经感染灶组织病理确诊，其中1例患儿经血、尿宏基因组二代测序（mNGS）检测确诊，后行感染灶（肾脏）切除病理检查结果与mNGS结果一致。2例患儿经肺泡灌洗液mNGS检测确诊。4例患儿为单部位感染，2例为播散性感染。6例患儿中5例有激素治疗史，1例曾接受造血干细胞移植。6例患儿发生感染时均有持续性中性粒细胞缺乏。感染类型包括肺毛霉病3例、鼻-鼻窦毛霉病1例、播散性毛霉病2例。

**表1:** 6例白血病合并毛霉病患儿临床资料

例号	年龄（岁）	性别	原发病	临床表现	既往治疗	感染部位	影像学	病原学	治疗药物	手术	转归
1	5	男	B-ALL	发热、鼻塞、流脓涕	化疗（激素）	鼻-鼻窦	鼻窦MRI：双侧上颌窦未充气，可见少低信号弥漫填充窦腔。窦壁可见黏膜强化	鼻甲黏膜组织病理	LAmB、泊沙康唑	鼻内病损切除术	完全缓解，存活
2	14	男	AML-M_2_	发热、踝部肿痛	化疗	皮肤软组织、肺	肺CT：右肺下叶实变，肺不张	踝部软组织、肺组织病理	LAmB、泊沙康唑	右肺中下叶切除术	部分缓解，存活
3	4	男	AML-M_7_	发热、咳嗽	化疗、移植	肺	肺CT：右肺上叶斑片致密影，内见支气管充气相。实变中心见相对低密度区，呈反晕征	肺组织病理	LAmB、泊沙康唑、AmB雾化	右上肺叶切除术	部分缓解，死于原发病复发
4	6	女	T-ALL	发热、咳嗽、咯血	化疗（激素）	肺	肺CT：右肺实变	肺泡灌洗液mNGS：微小根毛霉	LAmB、泊沙康唑、AmB雾化	右肺切除术	部分缓解，死于移植并发症
5	7	男	伯基特白血病	发热	化疗（激素）	肾脏、肺	肺CT：左肺下叶胸膜下结节肾MRI：右肾肿胀，实质内可见片状高信号区	尿、血mNGS：伞枝横梗霉；肾组织病理	LAmB、泊沙康唑	右肾切除术	完全缓解，存活
6	1	女	B-ALL	发热、咳嗽、气促	化疗（激素）	肺	肺CT：左肺上叶斑片致密灶伴支气管充气征，右肺上叶、中叶多个结节	肺泡灌洗液mNGS：米赫根毛霉	LAmB、泊沙康唑、AmB雾化	无	部分缓解，存活

注 B-ALL：急性B淋巴细胞白血病；AML：急性髓系白血病；T-ALL：急性T淋巴细胞白血病；LAmB：脂质体两性霉素B；AmB：两性霉素B；mNGS：宏基因组二代测序

二、辅助检查

1. 影像学：6例患儿均在确诊前进行影像学检查。其中肺部CT表现为肺不张、肺实变、结节影、支气管充气征、反晕征（图1）等。1例鼻-鼻窦毛霉病患儿鼻窦MRI表现为轴位T1WI可见少低信号弥漫填充窦腔；轴位、冠状位增强T1WI图像可见窦壁黏膜强化，窦腔区未见强化（图2）；鼻腔内镜：鼻中隔软骨裸露，黏膜糜烂，可见黑痂。1例播散性毛霉病肾脏MRI表现为轴位T1WI可见右肾形态饱满，实质信号欠均匀；轴位T2WI压脂序列可见右肾肿胀，实质内可见片状高信号区；冠状位T2WI压脂序列可显示病变位于右肾上极（图3）。

**图1:**
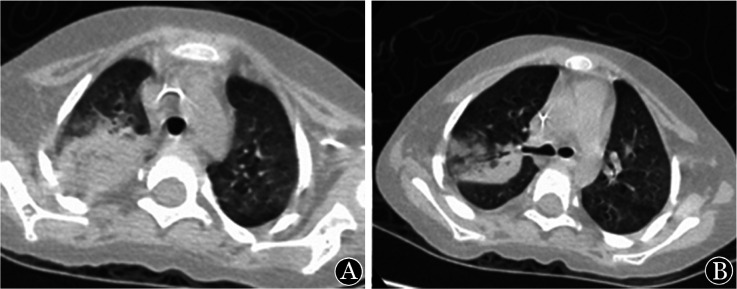
例3肺部CT A 右上肺大片致密影，中心部分的密度相对较低，呈反晕征； B 右上肺实变致密影和片影，可见支气管充气相

**图2:**
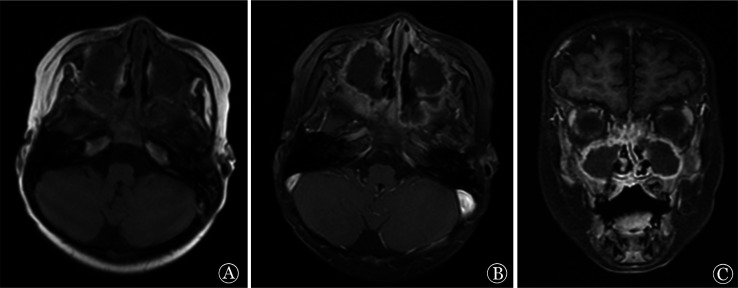
例1鼻窦部MRI A 轴位T1WI可见双侧上颌窦未充气，可见少低信号弥漫填充窦腔； B、C 轴位、冠状位增强T1WI图像可见窦壁黏膜强化，窦腔区未见强化

**图3:**
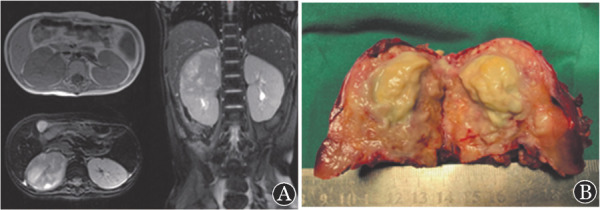
例5腹部MRI及病灶 A 腹部MRI轴位T1WI可见右肾形态饱满，实质信号欠均匀；轴位T2WI压脂序列可见右肾肿胀，实质内可见片状高信号区；冠状位T2WI压脂序列可显示病变位于右肾上极；左肾形态与信号未见异常。 B 右肾脓肿灶

2. 病原学：4例行病变组织病理检查：镜下均可见数量不等真菌菌团，菌丝较宽，无分隔，成钝角或直角分支的毛霉菌菌丝（图4），伴浸润组织脓肿、坏死、结构破坏，肉芽肿性炎症等表现。3例行mNGS检测，其中2例送检物为支气管灌洗液，1例送检物为外周血及中段尿，检测出病原微小根毛霉、米赫根毛霉、伞枝横梗霉各1例。

**图4:**
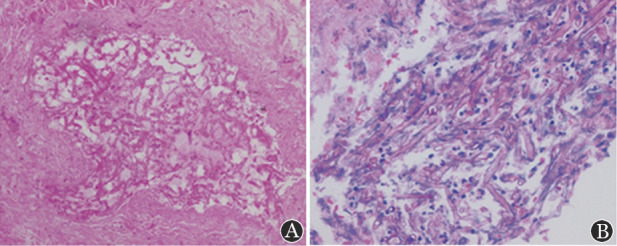
例2肺组织病理（HE染色） A 肉芽肿性炎，中央坏死物中见真菌丝（×200）； B 真菌菌团，菌丝较宽，无分隔，成钝角或直角分支的毛霉菌菌丝（×400）

三、治疗

6例患儿确诊后均给予脂质体两性霉素B静脉滴注治疗，疗程2～3周，3例肺部毛霉病患儿同时给予两性霉素B雾化。脂质体两性霉素B剂量为3～4 mg·kg^−1^·d^−1^，逐渐加量至6 mg·kg^−1^·d^−1^。两性霉素B雾化吸入剂量：浓度0.01％～0.02％，每次5～10 ml，每日2～3次。脂质体两性霉素静脉滴注期间，6例患儿均出现低钾血症，4例为轻至中度低钾血症（血钾2.5～3.5 mmol/L），2例为重度低钾血症（血钾低于2.5 mmol/L）；1例患儿出现间断高热。上述表现均于停药后缓解。后序贯泊沙康唑口服治疗，剂量：<34 kg者6 mg·kg^−1^·次^−1^，每日3次；≥34 kg者200 mg/次，每日4次。均在接受化疗或造血干细胞移植治疗期间持续口服，并监测泊沙康唑血药浓度（维持血药浓度≥1.0 µg/ml），2例患儿感染完全缓解并于维持治疗结束后停用。6例患儿中5例接受了感染灶切除手术治疗：1例皮肤软组织及肺部播散性毛霉病患儿接受踝部软组织清创术及右肺中下叶切除术；1例肾脏及肺部播散性毛霉病患儿接受右肾切除术，肺部感染灶经抗感染治疗后消失；1例鼻-鼻窦毛霉病患儿行鼻腔病灶切除术；2例肺部毛霉病患儿，1例因“大咯血”行紧急右肺全切术，1例行右肺上叶切除术。

四、转归

6例患儿在接受治疗后，2例感染完全缓解，4例感染部分缓解。4例存活，2例死亡。2例死亡患儿均在接受抗感染治疗及感染灶切除后部分缓解，但1例死于白血病复发，1例死于移植并发症。

## 讨论

毛霉菌感染较为罕见，发病率低于曲霉菌及念珠菌感染，为引起侵袭性真菌病的第三大病原，临床中较常见为根霉属（Rhizopus）、毛霉属（Mucor）及横梗霉属（Lichtheimia）所致的毛霉病[4]。经气道吸入毛霉菌引起的肺毛霉病在临床中最多见，其他较多见的原发感染部位为皮肤软组织、鼻窦及鼻-脑部等。毛霉病进展迅速，死亡率高。据文献报道，儿童血液肿瘤合并毛霉病死亡率为（39±8）％[5]。播散性毛霉病，特别是出现中枢神经系统感染时，死亡率可达80％以上[6]。早期识别、早期诊断、及时给予有效药物治疗，并积极进行手术清除感染灶等多学科治疗，可有效提高生存率。儿童发生毛霉病的危险因素包括：中性粒细胞缺乏、急性白血病、造血干细胞移植史、免疫缺陷、皮质类固醇激素治疗史、糖尿病、血液学复发恶性肿瘤、既往抗真菌药物预防史、年龄>10岁、早产、铁过载等[7]–[9]。本组6例患儿，原发病均为急性白血病，1例为复发且曾接受造血干细胞移植，发生感染时均处于粒细胞缺乏期，5例有糖皮质激素治疗史，均有抗真菌药物预防史，以上特点均符合毛霉病发生的危险因素。

毛霉病的诊断依赖于影像学及病原学。确诊金标准为病原学检查，包括组织病理、培养、分子检测等。毛霉菌在组织中典型表现为宽大、不规则，无分隔或极少分隔或直角分枝的菌丝。由于毛霉菌菌丝脆弱，且取材过程中极易被破坏，故培养阳性率极低 [10]。G、GM试验在毛霉菌感染时均呈阴性，故不适用于诊断本病。毛霉目特异性PCR检测具有较高的敏感性和特异性，但能否进行分子检测很大程度上取决于用于提取核酸和进行 PCR 测试的平台和试剂[11]，故无法广泛开展。基于上述原因，儿童毛霉病很难通过传统检测手段确诊。mNGS为近年来新开展的病原检测技术，具有无偏倚性、覆盖广、敏感性高、用时相对较短等优点，尤其可在混合感染或病毒感染方面展现其优势。国内相关指南推荐[12]，对于临床疑似感染的病危、病重或免疫抑制、免疫缺陷患者，常规检测无法确定病原或（和）规范抗感染治疗无效时，建议在完善传统实验室及分子生物学检测的同时，采集感染部位的标本进行mNGS检测。本研究中两例患儿经支气管肺泡灌洗液mNGS确诊，1例经尿mNGS确诊且后期行肾脏病理检测结果亦证实毛霉菌感染。本研究中6例患儿从出现症状至确诊时间为10～25 d，平均17 d。1例造血干细胞移植后复发的急性巨核细胞白血病患儿确诊时间为25 d，因其感染较重无法耐受手术，故先予脂质体两性霉素B治疗，待病情稳定后行手术切除肺部感染灶经病理确诊。对于病情不稳定或不能耐受手术而无法获得组织标本者，建议可选择肺泡灌洗液、外周血、尿液等进行mNGS以提高诊断速度及准确率，避免治疗延误。

确诊毛霉病后，应尽早进行有效抗真菌治疗并行感染灶切除，为目前普遍推荐的治疗策略。据报道，接受抗感染联合手术治疗可将儿童毛霉病的病死率由60％～68％降至11％～18.5％[13]–[14]，故手术清除感染灶为毛霉病治疗成功的关键要素之一。早期手术干预优于晚期手术，合理有效进行手术切除不仅可改善毛霉病的生存率，亦可减少健康组织不必要的损失[4]。脂质体两性霉素B或两性霉素B脂质体复合物为各年龄组儿童毛霉病的一线推荐治疗药物，尤其推荐用于治疗中枢神经系统感染。一般推荐剂量为5～<10 mg·kg^−1^·d^−1^，存在中枢神经系统感染时可予10 mg·kg^−1^·d^−1^ [15]。两性霉素B脱氧胆酸盐可用于当脂质体两性霉素B或两性霉素B脂质体复合物不可及时的治疗选择，但不良反应相对较大，需谨慎选择，推荐剂量为1～1.5 mg·kg^−1^·d^−1^。泊沙康唑和艾沙康唑推荐用于挽救或维持治疗。对于复杂难治性感染，可采用联合用药进行挽救治疗，建议两性霉素B脂质体复合物联合卡泊芬净，或两性霉素B脂质体复合物联合泊沙康唑。关于抗感染疗程，推荐两性霉素B治疗3周后予泊沙康唑口服维持治疗[16]，维持治疗疗程应根据患儿个体情况决定,一般持续数月至数年，强烈推荐治疗至免疫抑制状态逆转且影像学完全恢复[1]。研究证实，两性霉素B雾化吸入是血液肿瘤及造血干细胞移植患者肺部真菌病的有效治疗手段，且无严重不良反应，可在全身抗菌治疗基础上应用[17]。本研究中6例患儿，确诊后均使用脂质体两性霉素B静脉滴注治疗，依个体耐受情况疗程2～3周，后序贯泊沙康唑口服维持治疗。3例肺部毛霉病患儿接受两性霉素B雾化治疗。5例患儿接受感染灶切除手术。全部患儿感染均得到有效控制，4例存活且2例感染完全缓解，2例播散性感染患儿均存活且1例完全缓解。

儿童急性白血病合并毛霉病较为罕见，本病进展迅速，预后差。早期识别、快速诊断、合理抗感染、手术清除感染灶是治疗成功的关键要素。发生感染后，应有效控制感染，保障原发病治疗，以改善儿童白血病预后。
